# Mechanism of Secondary Glaucoma Development in HTLV-1 Uveitis

**DOI:** 10.3389/fmicb.2022.738742

**Published:** 2022-06-02

**Authors:** Yuan Zong, Koju Kamoi, Naoko Ando, Hisako Kurozumi-Karube, Kyoko Ohno-Matsui

**Affiliations:** Department of Ophthalmology and Visual Science, Graduate School of Medical and Dental Sciences, Tokyo Medical and Dental University, Tokyo, Japan

**Keywords:** human T-cell leukemia virus type 1, HTLV-1 uveitis, ocular inflammation, uveitis, glaucoma, ocular hypertension, NF- kappa B

## Abstract

Human T-cell lymphotropic virus type 1 (HTLV-1) was the first retrovirus identified as the causative agent of human diseases, such as adult T-cell leukemia, HTLV-1-associated myelopathy, and HTLV-1 uveitis (HU). HU is one of the most frequent ocular inflammatory diseases in endemic areas, which has raised considerable public health concerns. Approximately 30% of HU patients develop secondary glaucoma, which is higher than the general uveitis incidence. We therefore investigated the mechanism underlying the high incidence of glaucoma secondary to HU *in vitro.* After contact with HTLV-1-producing T cells (MT-2), human trabecular meshwork cells (HTMCs) were infected. The infected cells increased in number, and nuclear factor (NF)-κB expression was activated. Contact between MT-2 cells and HTMCs resulted in significantly upregulated production of inflammatory cytokines, such as IL-6, and chemokines, such as CXCL10, CCL2, and CXCL-8. These findings indicate that the mechanism underlying secondary glaucoma in HU may involve proliferation of trabecular meshwork tissue after contact with HTLV-1-infected cells, resulting in decreased aqueous humor outflow. Upregulated production of inflammatory cytokines and chemokines simultaneously disrupts the normal trabecular meshwork function. This mechanism presumably leads to increased intraocular pressure, eventually resulting in secondary glaucoma.

## Introduction

Human T-lymphotropic virus type 1 (HTLV-1) was the first retrovirus found to be infectious and pathogenic in humans (Miyoshi et al., 1981; Kamoi et al., 2018). HTLV-1 has been reported in many regions of the world, and it is highly endemic in the Caribbean islands, parts of central Africa, and Japan (Martin et al., 2018; Kamoi, 2020). HTLV-1 causes adult T-cell lymphoma, HTLV-1-associated myelopathy, and HTLV-1 uveitis (HU; Tagaya et al., 2019; Yamauchi et al., 2021). In areas in which HTLV-1 is highly endemic, HU is one of the most common ocular inflammatory diseases (Terada et al., 2017a; Kamoi et al., 2022b), which has raised considerable public health concerns (Kamoi and Mochizuki, 2012b; Kamoi, 2020; Kamoi et al., 2020, 2022a). Recent research indicates that horizontal transmission of HTLV-1 is responsible for HU, which raises concerns for populations in metropolitan areas (Kamoi et al., 2021, 2022b). The pathogenesis of HU has been described as involving the continuous accumulation in the eye of inflammatory cytokines produced by ocular-infiltrating HTLV-1-infected T cells, leading to inflammation of the eye (Kamoi and Mochizuki, 2012a). Ocular inflammation often leads to secondary ocular complications, such as secondary glaucoma, a sight-threatening condition associated with damage to the optic nerve, and subsequent loss of visual field due to elevated intraocular pressure (IOP; Kingman, 2004).

According to various surveys, approximately 30% of HU patients develop secondary glaucoma (Rathsam-Pinheiro et al., 2009; Terada et al., 2017a), a higher proportion compared to previous reports of the general incidence of uveitis (10–23%; Mercieca et al., 2017; Kesav et al., 2020). HU patients are thus more susceptible to secondary glaucoma. HTLV-1-infected cells in the aqueous humor are thought to adversely affect the trabecular meshwork, leading to an increase in ocular pressure. Therefore, in this study, we investigated the effect of HTLV-1-infected cells on the trabecular meshwork *in vitro*. We focused on human trabecular meshwork cells (HTMCs) and HTLV-1-infected MT-2 cells and analyzed immunologic/pathologic changes that contribute to increased ocular pressure in HU patients.

## Materials and Methods

### Cell Lines and Culture

HTMCs were purchased from Sciencell Research Laboratories (San Diego, CA, United States) and authenticated by testing the responsiveness of myocilin expression to treatment with dexamethasone (Yarishkin et al., 2019). HTMCs were isolated from the juxtacanalicular and corneoscleral regions of human eyes. MT-2 cells were adopted as model HTLV-1-infected T cells (Miyoshi et al., 1981; Yoshida et al., 1982), and Jurkat cells were used as HTLV-1-negative T cells as the control group (Uchida et al., 2019; Yarishkin et al., 2019; Kurozumi-Karube et al., 2020). Co-cultured T cells were irradiated with 9,000 rads in all but cytometric beads assay. HTMCs were cultured in Trabecular Meshwork Cell Medium (ScienCell Research Laboratories, Carlsbad, CA), which consisted of a proprietary basal medium formulation supplemented with 2% fetal bovine serum (FBS; GE Healthcare Japan, Tokyo, Japan), 1% fibroblast growth supplement, and 1% penicillin/streptomycin. MT-2 and Jurkat cells were cultured in RPMI 1640 (Wako Pure Chemical Corp., Osaka, Japan) supplemented with 10% FBS and 1% penicillin/streptomycin. All cells were cultured at 37°C in a humidified atmosphere containing 5% CO_2_. Cells were used for experiments at the third to fourth passages.

### Cell Infection

For HTMCs used for enumeration, HTLV-1 proviral load (PVL) measurement, and nuclear factor (NF)-κB activity ELISA, we used the standard method for HTLV-1 infection *in vitro* (Miyoshi et al., 1981; Akagi et al., 1986; Liu et al., 2006). Briefly, HTMCs were plated and co-cultured with three times the number of irradiated (9,000 rads) MT-2 or Jurkat cells for 48 h. The MT-2/Jurkat cells were then removed, and the attached HTMCs were transferred three times.

### Enumeration of HTMCs

As described above, after cell infection, HTMCs were transferred three times, detached using trypsin, and counted under a light microscope.

### Measurement of HTLV-1 PVL

According to the manufacturer’s instructions, an EZ1 Virus Mini kit v2.0 (Qiagen, Hilden, Germany) was used to prepare DNA from each sample. The HTLV-1 PVL in HTMCs was measured using quantitative real-time polymerase chain reaction (PCR), as described previously (Fukui et al., 2017; Uchida et al., 2019; Kurozumi-Karube et al., 2020). PVL was quantified using the HTLV-1 Tax primer (forward, 5′-CCCACTTCCCAGGGTTTGGA-3′; reverse, 5′-GGCCAGTAGGGCGTGA-3′) and probe (5′-FAM- CCAGTCTACGTGTTTGGA GACTGTGTACA-TAMRA-3′). Glyceraldehyde-3-phosphate dehydrogenase was used as the internal control.

### NF-κB Activity ELISA

The level of NF-κB phosphorylation in HTMCs infected with HTLV-1 was measured using an InstantOne ELISA kit [cat. no. 85-86083-11. eBioscience, CA (Consider also indicating the city), United States] according to the manufacturer’s instructions. Absorbance was read at 450 nm.

### Cytometric Beads Assay

For samples used for cytometric beads assays, HTMCs (1.5 × 10^5^ cells/ml) were allowed to adhere to 6-well plates overnight. Co-cultivation was carried out separately with and without the Transwell system. When using the Transwell system, HTMCs were cultivated in the lower chamber, and MT-2/Jurkat (5 × 10^5^ cells/ml) cells were seeded into the Transwell membrane of the upper chamber with 0.4 μm pore size. In cultures without the Transwell system, HTMCs and MT-2/Jurkat (5 × 10^5^ cells/ml) cells were co-cultivated in direct contact. The co-cultivation lasted 48 h. After co-cultivation, the culture supernatants were collected and stored at −80°C until assayed.

Levels of specific chemokines and cytokines in culture supernatants were measured using CBA Human Inflammation Cytokine kits according to the manufacturer’s guidelines (BD Biosciences, San Jose, CA). Data were analyzed using FCAP Array software, version 3.0 (BD Biosciences) according to the manufacturer’s instructions. Cytokines measured included IL-12p70, TNF-α, IL-10, IL-6, IL-1β, and IL-8, and the chemokines included CCL2, CCL5, CXCL8 (IL-8), CXCL9, and CXCL10.

### HTMC Culture With Increasing Concentrations of Cytokines and Chemokines

To determine whether changes in HTMCs co-cultured with MT-2 cells were related to increases in cytokine and chemokine levels after co-culture, HTMCs (3 × 10^3^) were cultured alone in 96-well plates. Cytokines and chemokines were then added at levels corresponding to the respective levels observed in co-culture. For the cytokine group: 30 ng/ml IL-6 was added; for the chemokine group, 40 ng/ml CCL-2, 0.5 ng/ml CXCL-10, and 25 ng/ml CXCL-8 were added based on our previous data. After 48 h of culture, HTMCs were detached using trypsin and counted under a light microscope.

### Statistical Analysis

Data are presented as the mean ± standard error of the mean. Statistical analyses were performed using SPSS software (ver. 18.0 for Windows; IBM Corp.). Differences in levels of cytokines and chemokines were analyzed using the unpaired Student’s *t*-test (with or without Welch’s correction). Values of *p* < 0.05 were considered significant.

## Results

### Detection of HTLV-1 Proviral DNA in HTMCs After Contact With HTLV-1-Infected Cells

To determine whether HTMCs were infected with HTLV-1, the presence of proviral DNA sequences of Tax was examined using real-time PCR. As described in the Materials and Methods section, MT2 cells and Jurkat cells were irradiated with 9,000 rads, which was reported as a lethal level for all treated cells (Liu et al., 2006). In our experiment, we confirmed that irradiated MT2 and Jurkat cells were not viable using trypan blue staining 10 days after irradiation. Repeated washing ensured that no irradiated MT2 cells persisted in the HTMC culture at the time of DNA isolation.

**Figure 1:**
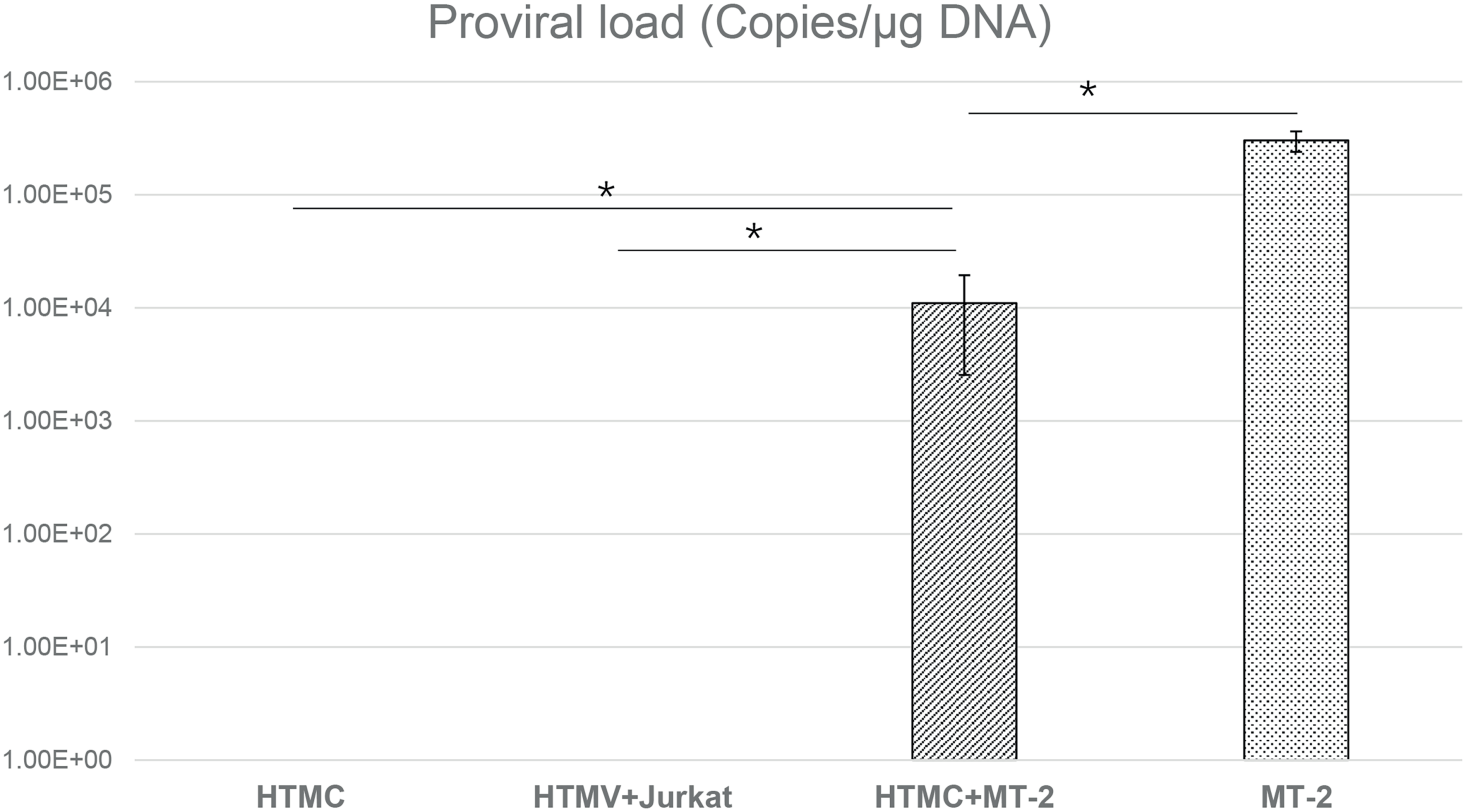
Detection of HTLV-1 proviral DNA by RT-PCR in HTMCs transferred three times after co-culture with MT-2 cells, Jurkat cells, or culture alone. The number of each respective cell type was 1 × 10^5^. The same number of MT-2 cells was used as a positive control. Data are taken from three independent biological experiments. Error bars represent standard deviation (^*^p < 0.05; n.s., not significant).

As shown in Figure 1, PVL was undetectable in analysis of DNA extracted from HTMCs co-cultured with irradiated Jurkat cells or HTMCs cultured alone as negative controls. In the positive control, PVL was detected in un-irradiated MT-2 cells. In DNA samples extracted from HTMCs co-cultured with irradiated MT-2 cells, PVL was detected, indicating that HTMCs were infected with HTLV-1 (Figure 1).

### Changes in the Number of Trabecular Meshwork Cells Following Contact With HTLV-1-Infected Cells

**Figure 2:**
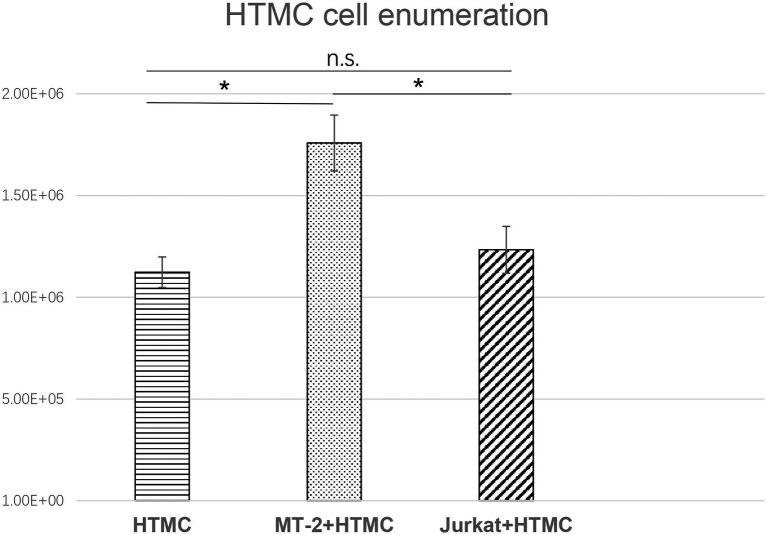
Enumeration of HTMCs co-cultured with irradiated Jurkat cells or cultured alone per well after three transfers. The number of HTMCs at the beginning of each culture was 1.5 × 10^5^. Compared with HTMCs co-cultured with irradiated Jurkat cells or cultured alone, the number of HTMCs co-cultured with irradiated MT-2 cells was significantly increased. Data represent three independent experiments, and average values are plotted. Error bars represent standard deviation (^*^p < 0.05; n.s., not significant).

To avoid adhesion of HTMCs to T cells, HTMCs co-cultured with irradiated MT-2/Jurkat cells were enumerated after three transfers. Compared with cells co-cultured with irradiated Jurkat cells or cultured alone, the number of HTMCs co-cultured with irradiated MT-2 cells increased significantly. HTMCs co-cultured with irradiated Jurkat cells tended to be slightly more numerous than HTMCs cultured alone, but no significant differences were found (Figure 2).

### Changes in Inflammatory Cytokine and Chemokine Levels in HTMCs Co-cultured With MT-2 Cells

**Figure 3:**
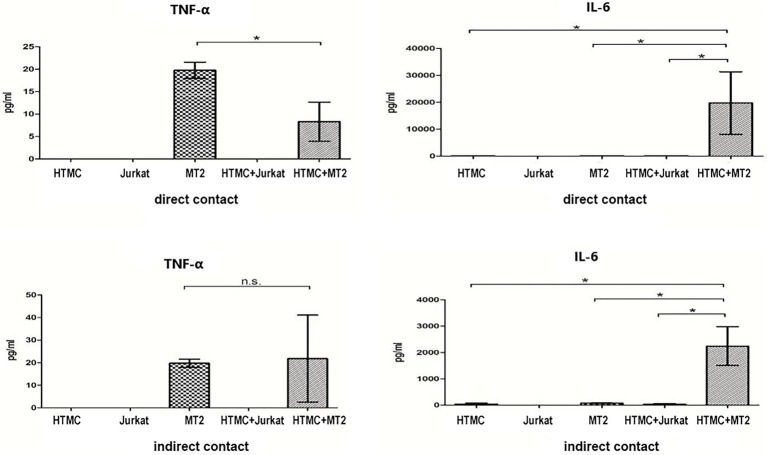
Inflammatory cytokines secreted by HTMCs, MT-2 cells, Jurkat cells, and HTMCs co-cultured with MT-2 or Jurkat cells under conditions of direct/indirect contact for 48 h. After co-culture with MT-2 cells, secretion of the cytokine IL-6 increased significantly. Data are taken from three independent biological experiments. Error bars represent standard deviation (units: pg./μl; ^*^p < 0.05; n.s., not significant).

**Figure 4:**
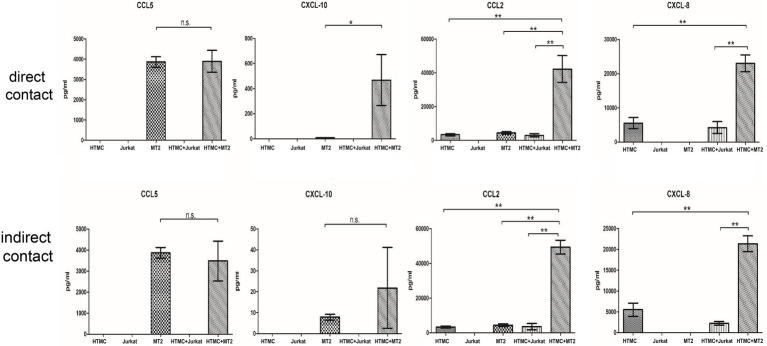
Chemokines secreted by HTMCs, MT-2 cells, Jurkat cells, and HTMCs co-cultured with MT-2 or Jurkat cells under conditions of direct/indirect contact for 48 h. After co-culture with MT-2 cells, levels of the chemokines CCL10, CCL2, and CXCL8 increased significantly. After co-culture with MT-2 cells using the Transwell system, levels of the chemokines CXCL10, CXCL8 (IL-8), and CCL2 increased significantly. Data are taken from three independent biological experiments. Error bars represent standard deviation (units: pg./μl; ^*^p < 0.05; ^**^p < 0.01; n.s., not significant).

The levels of inflammatory cytokines and chemokines can reflect the intensity of localized inflammation in the eye (Weinstein and Pepple, 2018). Thus, we measured levels of various cytokines (IL-6, IL-8, IL-1β, IL-12p70, IL-10, and TNF-α) and chemokines (CCL5, CXCL-9, CXCL8, CCL2, and CXCL10) secreted by HTMCs, MT-2 cells, Jurkat cells, HTMCs co-cultured with MT-2 cells, and HTMCs co-cultured with Jurkat cells (Figures 3, 4).

MT-2 cells cultured alone spontaneously secreted the cytokines TNF-α and IL-6 and chemokines CCL5, CXCL10, and CCL2, whereas HTMCs cultured alone spontaneously secreted the cytokine IL-6 and chemokines CCL2 and CXCL8 (Figures 3, 4). Following co-culture using the direct contact system, levels of IL-6, CCL2, CXCL8, and CXCL10 were significantly increased after co-culture compared with MT-2 cells cultured alone (Figures 3, 4). For cells cultured using the Transwell system, compared to MT-2 cells cultured alone, levels of the cytokine IL-6 and chemokines CCL2 and CXCL8 were significantly higher after co-culture (Figures 3, 4). Under all conditions, the cytokines IL-12p70, IL-1β, and IL-10 and the chemokine CXCL9 were below the detection limits (data not shown).

### Changes in NF-κB Activation in HTMCs

**Figure 5:**
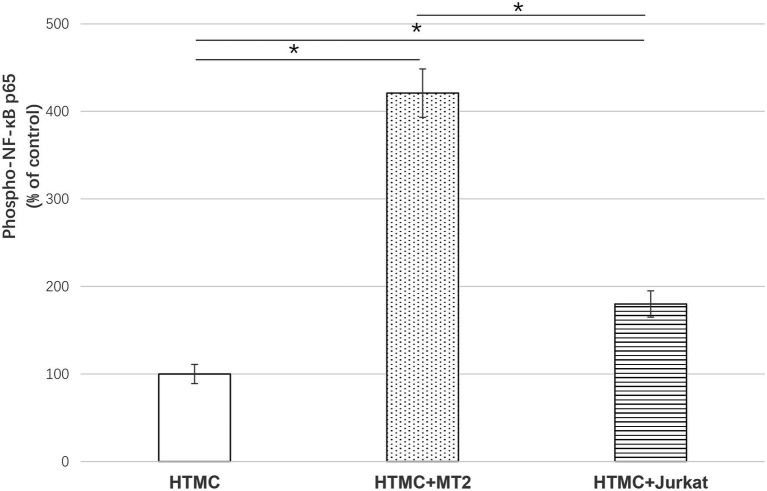
ELISA of phospho p65NFKB to show the effect of HTLV-1 infection on activation of NF-κB in HTMCs after three transfers. Data are expressed as percent of the control (%). Compared with HTMCs cultured alone, HTMCs co-cultured with irradiated MT-2 cells or irradiated Jurkat cells exhibited significantly increased phospho p65 NF-κB expression. Among the co-culture groups, HTMCs co-cultured with MT-2 cells exhibited significantly increased phospho p65NF-κB expression compared with HTMCs co-cultured with Jurkat cells. Error bars represent standard deviation (^*^p < 0.05; n.s., not significant).

To evaluate NF-κB activity, the expression of phospho p65 NF-κB was quantitatively estimated by ELISA. Compared with HTMCs cultured alone, HTMCs co-cultured with irradiated MT-2 or irradiated Jurkat cells exhibited significantly increased phospho p65 NF-κB expression (420.9 and 180.9% of the control, respectively, *p* < 0.001). Among the co-culture groups, phospho p65NF-κB expression was significantly increased in HTMCs co-cultured with MT-2 cells compared with HTMCs co-cultured with Jurkat cells (*p* < 0.05; Figure 5).

### Changes in the Number of HTMCs After Addition of Increasing Levels of Cytokines and Chemokines

**Figure 6:**
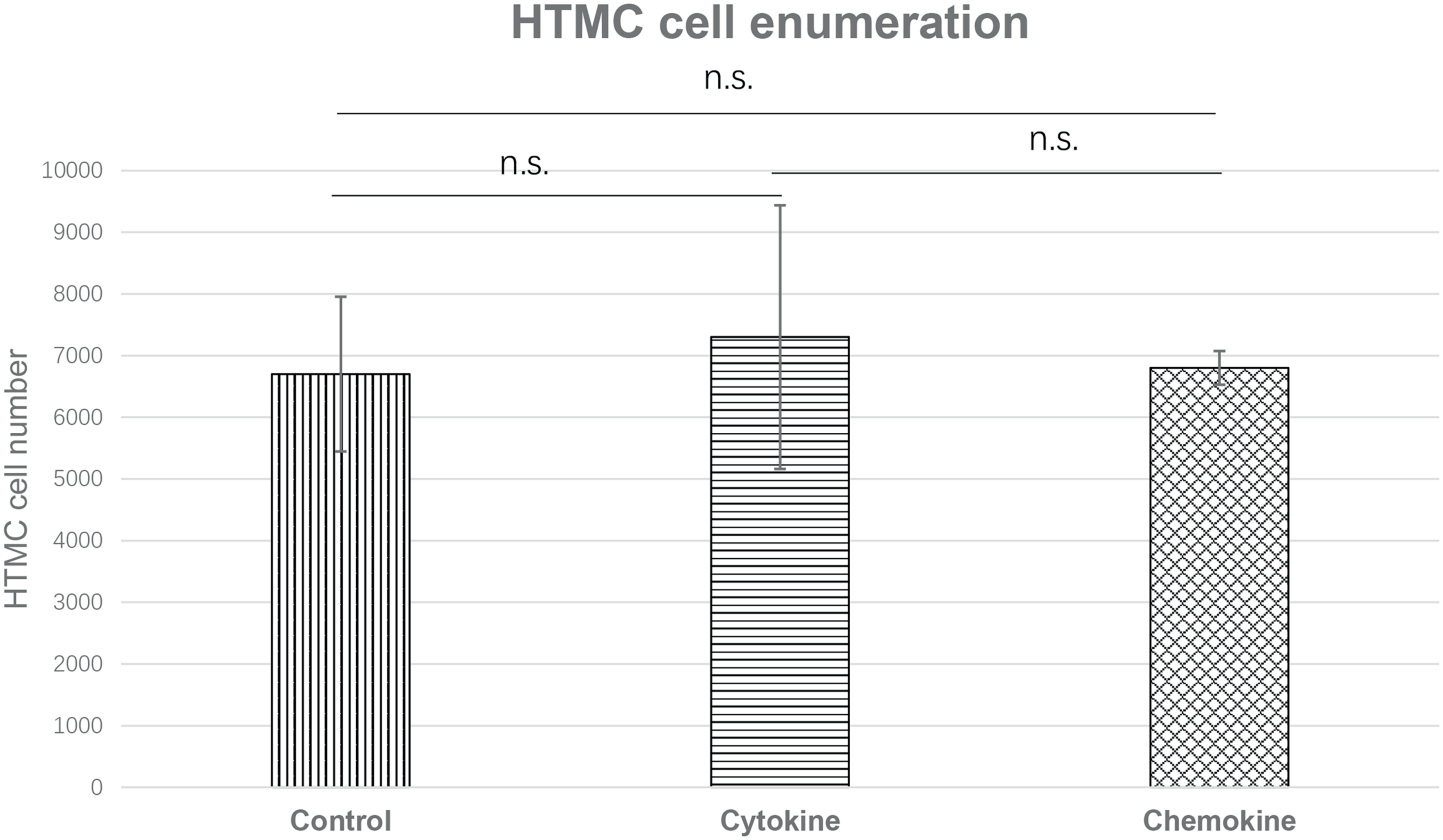
Enumeration of HTMCs cultured with increased concentrations of cytokine (IL-6 30 ng/ml) or chemokines (CCL-2 40 ng/ml, CXCL-10 0.5 ng/ml, and CXCL-8 25 ng/ml) for 48 h. The number of HTMCs was 5 × 10^3^ at the beginning of each respective culture. No significant changes in the number of HTMCs were detected among any groups.

We also examined whether the observed change in the proliferative capacity of HTMCs was associated with increased secretion of cytokines or chemokines. For the cytokine group: 30 ng/ml IL-6 was added, and for chemokine group, 40 ng/ml CCL-2, 0.5 ng/ml CXCL-10, and 25 ng/ml CXCL-8 was added based on the results of earlier experiments (Figures 3, 4). After 48 h, HTMCs were enumerated. Compared with the control group, no significant changes were observed in the number of HTMCs after addition of the corresponding cytokines or chemokines (Figure 6).

## Discussion

One of the most critical causes of increased IOP in patients with uveitis is a physical obstruction of the trabecular meshwork as a result of inflammatory processes, which leads to increased resistance to the flow of aqueous humor (Panek et al., 1990; Kalogeropoulos and Sung, 2018).

In this study, we examined the mechanism underlying the increase in ocular pressure secondary to HU *in vitro*. HTMCs co-cultured in direct contact with HTLV-1-infected T cells became infected with HTLV-1 and exhibited an increased cell proliferation rate and increased phosphorylation of NF-κB. In addition, specific inflammatory cytokines and chemokines were produced following contact between HTLV-1-infected T cells and HTMCs. These phenomena are thought to contribute to secondary glaucoma in HU patients.

These infiltrating HTLV-1-infected cells come into contact with the trabecular meshwork *via* outflow of aqueous humor, which is thought to affect the trabecular meshwork function. In the present study, therefore, we focused on trabecular meshwork cells and HTLV-1-infected cells to establish a model of the intraocular environment of HU patients and determine whether secondary glaucoma in HU is caused by changes in trabecular meshwork cells in contact with HTLV-1-infected cells. Pathologic changes in the trabecular meshwork led to blockage of the aqueous humor pathway, which ultimately leads to increased IOP and glaucoma.

Data regarding the PVL of human tissues and cells *in vivo* and cells are scant. A previous investigation identified retinal pigment epithelium as a potential reservoir for HTLV-1 that contributes to the breakdown of the blood–ocular barrier, resulting in HU (Liu et al., 2006; Uchida et al., 2019; Kurozumi-Karube et al., 2020). The present study therefore examined whether trabecular meshwork cells also function as reservoirs in HTLV-1-related diseases. To ensure that co-cultured T-cell lines were completely removed, we used T cells exposed to 9,000 rads in our experiments. After three generations of passaging, we confirmed that no T-cell lines were present and determined the HTLV-1 proviral load in HTMCs. HTLV-1 proviral DNA was detected in trabecular meshwork cells cultured in contact with HTLV-1-infected cells (Figure 1), demonstrating that HTLV-1 can infect trabecular meshwork cells and indicating that these cells are potential reservoirs for HTLV-1.

In this study, due to the possibility of adhesion of HTMCs to T cells, we used irradiated T-cell lines and confirmed the absence of T cells by microscopy after three generations of culture passage and then counted the HTMCs. Our data demonstrated that direct contact between trabecular meshwork cells and HTLV-1-infected cells leads to significant increases in the proliferation rate (Figure 2) of trabecular meshwork cells, thus narrowing the spaces in the trabecular meshwork and disrupting the outflow of aqueous humor, breaking the normal dynamic equilibrium.

Inflammation of the trabecular meshwork, known as trabeculitis, can lead to increased IOP, and studies indicate that trabeculitis can play a critical role in the elevation of IOP in herpetic uveitis, another common form of viral uveitis (Kalogeropoulos and Sung, 2018). Therefore, in the next phase of our study, we focused on analyzing changes in the levels of cytokines and chemokines in HTLV-1-infected cells co-cultured with trabecular meshwork cells under direct or indirect exposure conditions to assess whether there is an increased likelihood of trabeculitis occurring in HU patients. Regarding the production of cytokines and chemokines by cells co-cultured under conditions of direct/indirect contact, IL-6 expression increased significantly (Figures 3, 4). Levels of CCL2, CXCL8, and CXCL10 were also increased (Figures 3, 4). After HTLV-1-infected cells encounter the trabecular meshwork, the secreted IL-6 induces local inflammation (Kamoi and Mochizuki, 2012a; Terada et al., 2017b). The correlation between the inflammatory response and the initiation and progression of glaucoma has been demonstrated (Wei et al., 2018; Atanasovska Velkovska et al., 2021). Secreted CCL2, CXCL8, and CXCL10 attract immune cells, such as neutrophils and monocytes in the aqueous humor to the trabecular meshwork, which further impedes the outflow of aqueous humor.

NF-kB is an active transcription factor involved in regulation of the expression of various inflammatory mediators and cytokine genes, making it one of the key factors in inflammatory diseases (Lambrou et al., 2020; Pai and Sukumar, 2020). Due to this strong correlation with inflammation and oxidative stress, NF-κB is considered to play an important role in the pathogenesis of glaucoma (Klettner et al., 2013; Vernazza et al., 2019). Previous studies showed markedly higher activation of NF-κB p65 in the corneal tissues of glaucoma mice (Wei et al., 2018; Lei and Zhao, 2019). In addition, the NF-κB-mediated inflammatory stress response might be a predictor of the expression of SELE, a glaucoma marker, in trabecular meshwork cells (Wei et al., 2018). Notably, in the development of HTLV-1-associated disease, regulation of NF-κB activity is also a major pathway through which HTLV-1 activates gene transcription in host cells (Fochi et al., 2018; Vicario et al., 2018). We observed upregulation of the inflammatory cytokine IL-6, which is highly correlated with NF-κB activation, in the co-culture of trabecular meshwork cells and HTLV-1-infected cells. Therefore, we investigated whether the activation of NF-κB phosphorylation in trabecular meshwork cells was affected after cell infection. HTMCs exhibited significantly increased phospho p65 NF-κB expression after co-culture with irradiated MT-2 or Jurkat cells (Figure 5), indicating that HTLV-1 infection leads to increased activation of NF-κB expression in HTMCs. Compared with HTMCs co-cultured with irradiated Jurkat cells, infection with HTLV-1 *via* co-culture with irradiated MT-2 cells significantly increased phospho p65 NF-κB expression in HTMCs (Figure 5), suggesting that HTLV-1 infection substantially upregulates the activation of NF-κB in trabecular meshwork cells, ultimately leading to increased secretion of inflammatory cytokines and enhancing the inflammatory response in the trabecular meshwork.

To further clarify the mechanism of HU secondary to glaucoma, we further confirmed whether the enhanced proliferation of HTMCs is due to upregulated cytokine and chemokine expression by culturing HTMCs alone with the addition of the corresponding increased levels of cytokines or chemokines. No significant change in the proliferation rate of trabecular meshwork cells was detected with the addition of cytokines or chemokines (Figure 6), suggesting that the proliferation of HTMCs is caused by contact with HTLV-1-infected cells or by infection with HTLV-1.

Some limitations of this study should be considered when interpreting the results. We investigated the changes that occur after contact between HTMCs and HTLV-1-infected cells. However, whether the cause of these changes is the infection of HTMCs by HTLV-1 or contact of HTMCs with HTLV-1-infected cells was not analyzed in detail. This issue will be the subject of our future studies. Secondary, although co-cultured T-cell lines were lethally irradiated, it remains possible that irradiated MT-2 cells might fuse to HTMCs before elimination of MT-2 cells. Additionally, the experiments were performed *in vitro* using cell co-culture. Therefore, it is difficult to determine whether infection of HTMCs with HTLV-1 was due to the artificial environment *in vitro*. In parallel with basic research, clinical long-term tracking investigations of patients with HU secondary to glaucoma might also be needed.

## Conclusion

We investigated the mechanism of secondary glaucoma in HU patients *in vitro*. Trabecular meshwork cells were infected with HTLV-1, which contributed to an accelerated proliferation rate and reduced aqueous outflow. The accumulation of various chemokines recruited a large number of inflammatory cells into the trabecular meshwork, further exacerbating the obstruction and ultimately disrupting the aqueous outflow pathway. At the same time, the enhanced activation of NF-κB in trabecular meshwork cells led to increased secretion of cytokines, such as IL-6 and an increased local inflammatory response in the trabecular meshwork. In addition, HTLV-1 infection of trabecular meshwork cells disrupted the function of the trabecular meshwork. Obstruction of the aqueous outflow pathway and the deterioration of the trabecular meshwork function prevented the outflow of aqueous fluid, leading to an increase in IOP and eventually to secondary glaucoma.

## Data Availability Statement

The original contributions presented in the study are included in the article/supplementary material, further inquiries can be directed to the corresponding author.

## Author Contributions

YZ performed the experiments and wrote the draft of the manuscript. KK designed the experiments, analyzed the data, and wrote the manuscript. NA and HK-K performed the experiments. KO-M contributed to the analysis and interpretation of data and assisted in the preparation of the manuscript. All authors contributed to the article and approved the submitted version.

## Funding

This work was supported by JSPS KAKENHI grant number JP 20K09824.

## Conflict of Interest

The authors declare that the research was conducted in the absence of any commercial or financial relationships that could be construed as a potential conflict of interest.

## Publisher’s Note

All claims expressed in this article are solely those of the authors and do not necessarily represent those of their affiliated organizations, or those of the publisher, the editors and the reviewers. Any product that may be evaluated in this article, or claim that may be made by its manufacturer, is not guaranteed or endorsed by the publisher.

## References

[ref1] Akagi T. Takata H. Ohtsuki Y. Takahashi K. Oka T. Yano S.. (1986). Transformation of hamster spleen lymphocytes by human T-cell leukemia virus type I. Int. J. Cancer 37, 775–779. doi: 10.1002/ijc.2910370520, PMID: 3009333

[ref2] Atanasovska Velkovska M. Goričar K. Blagus T. Dolžan V. Cvenkel B. (2021). Association of genetic polymorphisms in oxidative stress and inflammation pathways with glaucoma risk and phenotype. J. Clin. Med. 10:1148. doi: 10.3390/jcm10051148, PMID: 33803434 PMC7967191

[ref3] Fochi S. Mutascio S. Bertazzoni U. Zipeto D. Romanelli M. G. (2018). HTLV deregulation of the NF-kappaB pathway: an update on tax and antisense proteins role. Front. Microbiol. 9:285. doi: 10.3389/fmicb.2018.00285, PMID: 29515558 PMC5826390

[ref4] Fukui S. Nakamura H. Takahashi Y. Iwamoto N. Hasegawa H. Yanagihara K.. (2017). Tumor necrosis factor alpha inhibitors have no effect on a human T-lymphotropic virus type-I (HTLV-I)-infected cell line from patients with HTLV-I-associated myelopathy. BMC Immunol. 18, 1–11. doi: 10.1186/s12865-017-0191-2 28158970 PMC5292003

[ref5] Kalogeropoulos D. Sung V. C. (2018). Pathogenesis of uveitic glaucoma. J. Cur. Glaucoma Pract. 12, 125–138. doi: 10.5005/jp-journals-10078-1236 PMC6647826 31354205

[ref6] Kamoi K. (2020). HTLV-1 in ophthalmology. Front. Microbiol. 11:388. doi: 10.3389/fmicb.2020.00388, PMID: 32218778 PMC7078647

[ref7] Kamoi K. Horiguchi N. Kurozumi-Karube H. Hamaguchi I. Yamano Y. Uchimaru K.. (2021). Horizontal transmission of HTLV-1 causing uveitis. Lancet Infect. Dis. 21:578. doi: 10.1016/S1473-3099(21)00063-3, PMID: 33773136

[ref8] Kamoi K. Mochizuki M. (2012a). HTLV infection and the eye. Curr. Opin. Ophthalmol. 23, 557–561. doi: 10.1097/ICU.0b013e328358b9ec 23047174

[ref9] Kamoi K. Mochizuki M. (2012b). HTLV-1 uveitis. Front. Microbiol. 3:270. doi: 10.3389/fmicb.2012.00270, PMID: 22837757 PMC3403349

[ref10] Kamoi K. Okayama A. Izumo S. Hamaguchi I. Uchimaru K. Tojo A.. (2018). Adult T-cell leukemia/lymphoma-related ocular manifestations: analysis of the first large-scale Nationwide survey. Front. Microbiol. 9:3240. doi: 10.3389/fmicb.2018.03240, PMID: 30671044 PMC6331419

[ref11] Kamoi K. Okayama A. Izumo S. Hamaguchi I. Uchimaru K. Tojo A.. (2020). Tackling HTLV-1 infection in ophthalmology: a nationwide survey of ophthalmic care in an endemic country, Japan. Brit. J. Ophthalmol. 104, 1647–1651. doi: 10.1136/bjophthalmol-2019-315675, PMID: 32152142

[ref12] Kamoi K. Uchimaru K. Tojo A. Watanabe T. Ohno-Matsui K. (2022a). HTLV-1 uveitis and Graves' disease presenting with sudden onset of blurred vision. Lancet 399:60. doi: 10.1016/S0140-6736(21)02442-9, PMID: 34973718

[ref13] Kamoi K. Watanabe T. Uchimaru K. Okayama A. Kato S. Kawamata T.. (2022b). Updates on HTLV-1 uveitis. Viruses 14:794. doi: 10.3390/v14040794, PMID: 35458524 PMC9030471

[ref14] Kesav N. Palestine A. G. Kahook M. Y. Pantcheva M. B. (2020). Current management of uveitis-associated ocular hypertension and glaucoma. Surv. Ophthalmol. 65, 397–407. doi: 10.1016/j.survophthal.2019.12.003, PMID: 31816329

[ref15] Kingman S. (2004). Glaucoma is second leading cause of blindness globally. Bull. World Health Organ. 82, 887–888. 15640929 PMC2623060

[ref16] Klettner A. Westhues D. Lassen J. Bartsch S. Roider J. (2013). Regulation of constitutive vascular endothelial growth factor secretion in retinal pigment epithelium/choroid organ cultures: p38, nuclear factor κB, and the vascular endothelial growth factor receptor-2/phosphatidylinositol 3 kinase pathway. Mol. Vis. 19, 281–291. 23401656 PMC3566904

[ref17] Kurozumi-Karube H. Kamoi K. Ando N. Uchida M. Hamaguchi I. Ohno-Matsui K. (2020). In vitro evaluation of the safety of Adalimumab for the eye Under HTLV-1 infection status: A preliminary study. Front. Microbiol. 11:522579. doi: 10.3389/fmicb.2020.522579, PMID: 33424777 PMC7785715

[ref18] Lambrou G. I. Hatziagapiou K. Vlahopoulos S. (2020). Inflammation and tissue homeostasis: The NF-κB system in physiology and malignant progression. Mol. Biol. Rep. 47, 4047–4063. doi: 10.1007/s11033-020-05410-w, PMID: 32239468

[ref19] Lei X. Zhao Y. (2019). Neovascular glaucoma regulation by arylsulfonyl indoline-benzamide (ASIB) through targeting NF-kB signalling pathway. Biotech 9, 211–216. doi: 10.1007/s13205-019-1730-8, PMID: 31093481 PMC6510736

[ref20] Liu B. Li Z. Mahesh S. P. Kurup S. K. Giam C. Z. Nussenblatt R. B. (2006). HTLV-1 infection of human retinal pigment epithelial cells and inhibition of viral infection by an antibody to ICAM-1. Invest. Ophthalmol. Vis. Sci. 47, 1510–1515. doi: 10.1167/iovs.05-1277, PMID: 16565386

[ref21] Martin F. Tagaya Y. Gallo R. (2018). Time to eradicate HTLV-1: an open letter to WHO. Lancet 391, 1893–1894. doi: 10.1016/S0140-6736(18)30974-7, PMID: 29781438

[ref22] Mercieca K. Steeples L. Anand N. (2017). Deep sclerectomy for uveitic glaucoma: long-term outcomes. Eye 31, 1008–1019. doi: 10.1038/eye.2017.80, PMID: 28643797 PMC5519282

[ref23] Miyoshi I. Kubonishi I. Yoshimoto S. Akagi T. Ohtsuki Y. Shiraishi Y.. (1981). Type C virus particles in a cord T-cell line derived by co-cultivating normal human cord leukocytes and human leukaemic T cells. Nature 294, 770–771. doi: 10.1038/294770a0, PMID: 6275274

[ref24] Pai P. Sukumar S. (2020). HOX genes and the NF-κB pathway: A convergence of developmental biology, inflammation and cancer biology. Cancer 1874:188450. doi: 10.1016/j.bbcan.2020.188450 33049277

[ref25] Panek W. C. Holland G. N. Lee D. A. Christensen R. E. (1990). Glaucoma in patients with uveitis. Br. J. Ophthalmol. 74, 223–227. doi: 10.1136/bjo.74.4.223, PMID: 2337547 PMC1042066

[ref26] Rathsam-Pinheiro R. H. Boa-Sorte N. Castro-Lima-Vargens C. Pinheiro C. A. Castro-Lima H. Galvão-Castro B. (2009). Ocular lesions in HTLV-1 infected patients from Salvador, state of Bahia: the city with the highest prevalence of this infection in Brazil. Rev. Soc. Bras. Med. Trop. 42, 633–637. doi: 10.1590/S0037-86822009000600004, PMID: 20209345

[ref27] Tagaya Y. Matsuoka M. Gallo R. (2019). 40 years of the human T-cell leukemia virus: past, present, and future. F1000 Res. 8:228. doi: 10.12688/f1000research.17479.1, PMID: 30854194 PMC6396841

[ref28] Terada Y. Kamoi K. Komizo T. Miyata K. Mochizuki M. (2017a). Human T cell leukemia virus type 1 and eye diseases. J. Ocul. Pharmacol. Ther. 33, 216–223. doi: 10.1089/jop.2016.0124 28263674

[ref29] Terada Y. Kamoi K. Ohno-Matsui K. Miyata K. Yamano C. Coler-Reilly A.. (2017b). Treatment of rheumatoid arthritis with biologics may exacerbate HTLV-1-associated conditions: A case report. Medicine 96:e6021. doi: 10.1097/MD.0000000000006021, PMID: 28178142 PMC5312999

[ref30] Uchida M. Kamoi K. Ando N. Wei C. Karube H. Ohno-Matsui K. (2019). Safety of infliximab for the eye Under human T-cell leukemia virus type 1 infectious conditions in vitro. Front. Microbiol. 10:2148. doi: 10.3389/fmicb.2019.02148, PMID: 31620105 PMC6759608

[ref31] Vernazza S. Tirendi S. Scarfì S. Passalacqua M. Oddone F. Traverso C. E.. (2019). 2D-and 3D-cultures of human trabecular meshwork cells: A preliminary assessment of an in vitro model for glaucoma study. PLoS One 14:e0221942. doi: 10.1371/journal.pone.0221942, PMID: 31490976 PMC6731014

[ref32] Vicario M. Mattiolo A. Montini B. Piano M. A. Cavallari M. Amadori A.. (2018). A preclinical model for the ATLL lymphoma subtype With insights Into the role of microenvironment in HTLV-1-mediated Lymphomagenesis. Front. Microbiol. 9:1215. doi: 10.3389/fmicb.2018.01215, PMID: 29951044 PMC6008390

[ref33] Wei H. Y. Zhang Y. J. Zhao S. Z. (2018). Puerarin regulates neovascular glaucoma through pigment epithelium-derived growth factor-induced NF-κB signaling pathway. Mol. Med. Rep. 17, 7866–7874. doi: 10.3892/mmr.2018.8800, PMID: 29620183

[ref300] Weinstein J. E. Pepple K. L. (2018). Cytokines in uveitis. Curr. Opin. Ophthalmol 29, 267–274. doi: 10.1080/03014460500097237 29521875 PMC7199509

[ref34] Yamauchi J. Araya N. Yagishita N. Sato T. Yamano Y. (2021). An update on human T-cell leukemia virus type I (HTLV-1)-associated myelopathy/tropical spastic paraparesis (HAM/TSP) focusing on clinical and laboratory biomarkers. Pharmacol. Ther. 218:107669. doi: 10.1016/j.pharmthera.2020.107669, PMID: 32835825

[ref35] Yarishkin O. Phuong T. T. Križaj D. (2019). Trabecular meshwork TREK-1 channels function as polymodal integrators of pressure and pH. Invest. Ophthalmol. Vis. Sci. 60, 2294–2303. doi: 10.1167/iovs.19-26851, PMID: 31117121 PMC6532698

[ref36] Yoshida M. Miyoshi I. Hinuma Y. (1982). Isolation and characterization of retrovirus from cell lines of human adult T-cell leukemia and its implication in the disease. Proc. Natl. Acad. Sci. 79, 2031–2035. doi: 10.1073/pnas.79.6.2031, PMID: 6979048 PMC346116

